# Not every estimate counts – evaluation of cell composition estimation approaches in brain bulk tissue data

**DOI:** 10.1186/s13073-023-01195-2

**Published:** 2023-06-07

**Authors:** Lilah Toker, Gonzalo S. Nido, Charalampos Tzoulis

**Affiliations:** 1 Neuro-SysMed Center of Excellence, Department of Neurology, Department of Clinical Medicine, Haukeland University Hospital, University of Bergen, 5021 Bergen, Norway; 2 Department of Clinical Medicine, University of Bergen, Pb 7804, 5020 Bergen, Norway; 3 K.G Jebsen Center for Translational Research in Parkinson’s Disease, University of Bergen, Bergen, Norway

**Keywords:** Cell composition, Neurodegeneration, Omics, Deconvolution, Brain, Bulk tissue

## Abstract

**Background:**

Variation in cell composition can dramatically impact analyses in bulk tissue samples. A commonly employed approach to mitigate this issue is to adjust statistical models using estimates of cell abundance derived directly from omics data. While an arsenal of estimation methods exists, the applicability of these methods to brain tissue data and whether or not cell estimates can sufficiently account for confounding cellular composition has not been adequately assessed.

**Methods:**

We assessed the correspondence between different estimation methods based on transcriptomic (RNA sequencing, RNA-seq) and epigenomic (DNA methylation and histone acetylation) data from brain tissue samples of 49 individuals. We further evaluated the impact of different estimation approaches on the analysis of H3K27 acetylation chromatin immunoprecipitation sequencing (ChIP-seq) data from entorhinal cortex of individuals with Alzheimer’s disease and controls.

**Results:**

We show that even closely adjacent tissue samples from the same Brodmann area vary greatly in their cell composition. Comparison across different estimation methods indicates that while different estimation methods applied to the same data produce highly similar outcomes, there is a surprisingly low concordance between estimates based on different omics data modalities. Alarmingly, we show that cell type estimates may not always sufficiently account for confounding variation in cell composition.

**Conclusions:**

Our work indicates that cell composition estimation or direct quantification in one tissue sample should not be used as a proxy to the cellular composition of another tissue sample from the same brain region of an individual—even if the samples are directly adjacent. The highly similar outcomes observed among vastly different estimation methods, highlight the need for brain benchmark datasets and better validation approaches. Finally, unless validated through complementary experiments, the interpretation of analyses outcomes based on data confounded by cell composition should be done with great caution, and ideally avoided all together.

**Supplementary Information:**

The online version contains supplementary material available at 10.1186/s13073-023-01195-2.

## Background

Despite the rapid advance in single-cell technologies, bulk tissue samples remain the main source of data, especially in fields which require large numbers of samples, such as neurodevelopmental and neurodegenerative disorders of complex aetiology. This is particularly true for omics research beyond transcriptomics such as DNA methylation, histone modifications, and proteomics, where single-cell approaches are still in their infancy or do not yet exist. Bulk tissue data comes, however, with a major caveat of heterogeneity in the cellular composition of the samples being analysed. Accounting for this heterogeneity is absolutely essential for analysis and interpretation of bulk tissue data [1–6]. Indeed, over the last decade, multiple approaches have been developed to estimate the prevalence of different cell types directly from transcriptomic [7–11], DNA-methylation [3, 12, 13], and chromatin immunoprecipitation sequencing (ChIP-seq) [5] data. Cellularity estimates, which alone provide valuable information on the association between cellular composition and the condition of interest, can then be incorporated into statistical models used for data analysis [1, 4, 5, 14, 15] to adjust for the variation in cellular composition of the samples.

**Fig. 1:**
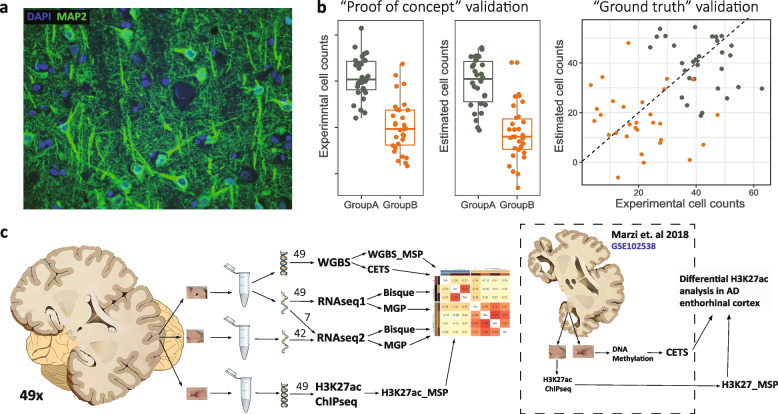
Study workflow. **a** IHC section in PFC showing the complexity of the human brain. DAPI staining (blue) was used to identify cell nuclei. MAP2 (green) is a neuronal marker expressed in neuronal bodies and processes. Neuronal processes present in the section may originate from the same cell as the nuclei, from nearby cells, or from projecting neurons whose bodies are located in an entirely different brain region. **b** The caveat of “proof of concept” validation for cell composition estimation methods. Simulated data, each point corresponds to one sample. The estimated counts (middle) show high performance based on “proof of concept” validation, recapitulating the group differences observed with experimental counts (left). However, direct comparison of the estimated and direct counts across samples (“Ground truth validation”) indicates poor correlation between the two, and failure of the estimates to correctly recapitulate the intra-group variability. **c** Study workflow. Performance of different estimation approaches was assessed through correlations among the estimates in same or nearby tissue samples from 49 individuals (left) and through re-analysis of H3K27ac ChIP-seq data with major differences in cell composition between the groups. Detailed description is provided under “Methods” section and Fig. 4a

While including cell count estimates as covariates in statistical models is becoming increasingly common, it is not always known to which extent these estimates recapitulate the actual cell composition of the samples, and whether this adjustment can sufficiently account for the across-sample variation in cell composition. Indeed, accurate assessment of the estimation methods requires knowledge of the ground truth with regard to the cellular composition of the samples. This requirement can be met for tissues where individual cells exist in suspension, such as blood, and samples can be aliquoted into fractions with identical cell composition - one for direct enumeration and the other for estimation [7, 13, 15]. The same approach, however, cannot be applied to brain tissue where cells are strongly interconnected with each other, sometimes over extended distances. For this reason, dissociation of entire brain cells, neurons in particular, is not feasible. Moreover, the specific conditions required for sample preparation for direct enumeration (e.g. immunohistochemistry staining) and omics analyses (e.g. transcriptomics), imply that the same tissue sample cannot be subjected to both procedures. Due to these technical limitations, the performance of the existing estimation methods in brain tissue has either not been assessed at all or evaluated exclusively using “proof of concept” approaches. These include (1) assessment of real/pseudo cell mixtures which lack the complexity of brain tissue (e.g. axonal and synaptic fractions, Fig. 1a), thus producing over-optimistic results [3, 11, 16], (2) comparing regions/conditions with known differences in cell composition [1, 3, 9], or (3) comparing the estimated abundances across cell types [10, 11, 15]. While these types of validation are acceptable during the initial steps of method development, they cannot reliably assess method performance in biological tissue samples, where the intra-individual differences are much more subtle and regulatory changes in individual molecules are likely to be involved (Fig. 1b).

The few attempts to directly compare brain tissue-based estimates to experimental cell counts [1, 15] used data from different tissue samples of the same individuals derived from different hemispheres. In such experimental design, however, the two tissue samples would necessarily exhibit very different tissue architectures, compromising the direct comparison. Furthermore, since in both studies the samples were obtained from two groups of individuals where cell composition is known to be associated with the disease, this approach for comparison is in fact very similar to the “proof of concept” validation (2). In other words, the very modest correlations observed between the estimates and the experimental cell counts (0.3–0.5) can be explained by the large inter-group variation, rather than interindividual variation inside each group [15]. Thus, to which extent the existing methods for cell type estimation can recapitulate the true cellular composition in human brain tissue remains unknown. Moreover, whether cell enumeration in one brain tissue sample can be used as a proxy for cellular composition of neighbouring tissue sample was never assessed.

In the current work, we assessed the correspondence between different estimation methods based on transcriptomics (RNA sequencing, RNA-seq), DNA methylation (whole genome bisulfite sequencing, WGBS), and H3K27 acetylation ChIP-seq (H3K27ac) data from prefrontal cortex (PFC) of 49 individuals. Cell composition was estimated for the five major brain cell types: neurons, astrocytes, microglia, oligodendrocytes and endothelial cells. The estimates were derived either from the same (RNA-seq and WGBS) or adjacent (RNA-seq, WGBS, H3K27ac) tissue samples. To gain further insight, we evaluated the impact of different estimation approaches on the analysis outcome of H3K27ac data from the entorhinal cortex of individuals with Alzheimer’s disease (AD) and controls, a brain area severely affected by neuronal loss already in the early stages of the disease. The outline of the work is shown in Fig. 1c, and the specific estimation approaches tested are described in Additional file 1: Table S1.

## Methods

For all omics modalities, the data was obtained from fresh-frozen prefrontal cortex (Brodmann area 9) samples of 49 individuals from two independent cohorts. The first cohort (Park West, PW), comprised individuals with idiopathic Parkinson’s disease (PD, *n* = 18) from a prospective population-based cohort which has been described in detail [17] and neurologically healthy controls (*n* = 11) from our brain bank for ageing and neurodegeneration. The second cohort comprised 21 individuals from the Netherlands Brain Bank (NBB) including idiopathic PD (*n* = 10) and demographically matched neurologically healthy controls (*n* = 11). The demographic information of the individuals, as well as RNA quality metrics and tissue sample characteristics, are provided in Additional file 1: Table S2 and Fig. 1c. ChIP-seq and WGBS data were obtained from our previously published work [1, 18]. The code to generate all the analyses and figures presented in this work can be accessed through the github repository: https://github.com/ltoker/Cellephant [19]. All the information regarding the version of the software and the packages used for the analysis are provided in the SessionInfo.Rds file.

### RNA-seq data

RNA sequencing was performed as previously described [2]. For 42/49 subjects, two different tissue samples were sent for sequencing, while the same tissue extract was sequenced twice for seven of the individuals (Fig. 1c, Additional file 1: Table S2). Data preprocessing was performed using the same pipeline as previously descibed [2], using the updated software and transcriptome versions. Specifically, transcript quantification was carried out using Salmon v1.3 [20] using the GENCODE reference annotation v35 [21].

### Calculation of Marker Gene Profiles (MGP)

Calculations were performed as previously described [1, 2]. For the purpose of this analysis, both cohorts were analysed together.

### Calculation of Bisque estimates

Bisque estimates were calculated using the “ReferenceBasedDecomposition” function from “BisqueRNA” R package (https://github.com/cozygene/bisque) [10], according to the author’s manual. Reference single set data were obtained from Darmanis et al. [22]. The “markers” variable was defined as the union between two sets of human brain cell type-specific marker genes, defined in Kelley et al. [23] and Velmeshev et al. [24] studies. Setting the “markers” variable to “NULL,” produced highly similar correlates (*r* > 0.98).

### Calculation of dtangle and CIBERSORT estimates

Estimates were obtained through https://voineagulab.shinyapps.io/BrainDeconvShiny/, a Shinyapp developed by Sutton et al. [11]. As input, we used counts per million (CPM) matrices. Cell type marker signature was selected as “MB,” since this signature was reported by the authors to produce the best outcomes for both methods.

### Calculation of Marker Site Profiles (MSP)

In order to identify brain cell type-specific H3K27ac regions, we first analysed H3K27ac ChIP-seq data from NeuN^+^ and NeuN^−^ brain cells [25]. Cell type-based broadPeaks (CellType_peak-set), BAM files and metadata files were downloaded from https://www.synapse.org/#!Synapse:syn5613802. Differential acetylated regions (DAR) between NeuN^+^ (neurons) and NeuN^−^ (glia) cells were calculated using “DESeq2” R package [26], including chromatin amount, library batch, sex, hemisphere, age and pH as covariates in the model. Peaks were defined as cell type-specific differentially acetylated regions (DARs) if they met the following criteria: (1) |fold change|> 4 and 2) mean count > 1000. Peaks were annotated to genes using the “build_annotations” function from “annotatr” R package based on UCSC hg19 genome assembly. Peaks were annotated to all genes for which they intersected a region between 5 kb upstream from their transcription start site (TSS) to the end of their 5′UTR. In the next step, we intersected the genes with DARs between glia and neurons with expression-based cortical marker gene lists based on NeuroExpresso database [9]. The DARs were next reassigned to specific glial and neuronal cells if they were annotated to genes defined as cell type-specific based on NeuroExpresso. For example, all DARs annotated to *MBP*, defined as an oligodendrocyte marker gene based on NeuroExpresso, were defined as oligodendrocyte marker sites (MSS). In the next step, reads based on BAM files from bulk tissue data were quantified in regions defined by the CellType_peak-set. The corresponding reads in peaks (RiP) were then converted to CPM and transformed to log2(CPM + 1). Next, for each cell type-specific MSS, we performed principal component analysis based on the relevant peaks using “prcomp” function from the “stats” R package using (scale = T), as described in [9]. Marker Site Profiles (MSP) were defined as the scores of the samples in the first principal component, transformed to [0,1] range for visualization purposes.

### Calculation of CETS estimates

CETS estimates were calculated as described in [3] using an implementation in R provided by the authors. CETS marks (genomic sites exhibiting differential methylation between neurons and glial cells) were lifted over from hg19 to hg38 using the R package “liftOver”, v1.18.0 [27].

### Calculation of WGBS_MSP

CETS marks [3] were assigned to specific cell types if they were located within regions annotated to expression-based cortical marker gene lists from NeuroExpresso database [9]. CETS marks mapped to multiple cell types or with coverage below 10 × in more than 20% of the samples were excluded. The final list of sites and their respective annotation to cell types is provided in Additional file 2. The methodology for calculating WGBS-MSP was analogous to that described for MSP/MGP but using the methylation levels in the cell type-categorized CETS marks. Briefly, for each cell type, the relevant methylation levels of CETS marks in all subjects were subjected to principal component analysis (missing values were estimated using the across-sample median). The corresponding scores of the samples in the first principal component were rescaled to [0,1], and the WGBS_MSPs for the tested cell type were then defined as 1-rescaled value.

### Correlation between estimation methods

Correlation values were obtained using Pearson’s correlation between pairs of estimates across individuals. The seven individuals resequenced twice for the transcriptomics analyses were excluded from the correlation analysis comparing all estimates together.

### Re-analysis of Marzi et al*.*

For the purpose of the re-analysis, we used the count matrix and BED files provided by the authors through GSE102538. We first reproduced the output of the analysis as described in the manuscript using the code provided by the authors and validated that the results are identical to those reported in the manuscript. For the purpose of the re-analysis using MSPs, the design formula of the original analysis ($\sim agef+ CETSif +condition$) was adjusted to either:

1 $$\sim agef+ Neuro{n}_{MSP}if +condition$$

or

2 $$\sim agef+ Neuro{n}_{MSP}+ Microgli{a}_{MSP}+{Oligo}_{MSP}+condition$$

where Neuron_*MSP*_if is neuronal MSP converted to a five-level ordered factor, to conform to the methodological approach in the original publication.

### Assessment of the case–control analysis output to H3K27ac in neurons and glial cells

BAM files of H3K27ac ChIP-seq data from NeuN^+^ and NeuN^−^ brain cells [25] and metadata files were downloaded from https://www.synapse.org/#!Synapse:syn5613802, and reads in peaks defined in BED files from Marzi et al. were quantified using “Rsubread” v2.0.2 R package [28]. In order to make the analysis more comparable with the case–control analysis from Marzi et al., differential H3K27ac analysis was performed using “edgeR” v3.28.1 R package [29], adjusting for sex, hemisphere, age and pH.

### Comparison of the case–control analysis output to brain cell type-specific enhancer and promoter regions

Cell type-specific enhancer and promoter regions defined in Nott et al. [30] were obtained through “echolocatoR” R package [31] github repository. Overlapping regions were identified using “findOverlaps” function from “IRanges” v2.28.0 R package [32], using the default parameters (> = 1 overlapping position).

## Results

### Different transcriptomics-based estimation methods produce highly correlated estimates of cellular composition

We first assessed the similarity between the outcomes of different transcriptomics-based estimation methods applied to brain bulk tissue data. For the purpose of this analysis, we leveraged two RNA-seq datasets from PFC tissue samples of 49 individuals, comprising Parkinson’s disease patients and controls (Fig. 1c, Additional file1: Table S2). We chose to focus on four estimation methods: Marker Gene Profiles (MGP) [9], CIBERSORT [7], dtangle [33], and Bisque [10]. These methods were selected because they have substantial differences in multiple parameters that can impact the performance of estimation approaches such as cell type markers, estimation algorithm and the type of outcome (Additional file1: Table S1), as well as publication date (2015–2020) and general popularity. CIBERSORT and dtangle were chosen specifically since they were reported to outperform other transcriptomics estimation approached in bulk brain tissue data in the recent work by Sutton et al. [11].

Despite the major methodological differences between the estimation methods, they produced highly correlated outcomes (Pearson’s correlation, interquartile range (IQR) 0.67 − 0.91). However, upon closer examination, the correlation values varied between cell types (Additional file1: Fig. S1a). While the median correlation between methods for neurons, astrocytes, and oligodendrocytes was ~ 0.9, substantially lower correlations were observed for microglia (*r* = 0.61) and endothelial cells (*r* = 0.57). The correlation with other methods was particularly low for CIBERSORT. To better understand the reason behind the low correlation, we compared the estimated proportion of each cell type to immunohistochemistry (IHC)-based cellular proportions in the PFC, reported in Patrick et al [15]. While these are not the same individuals and thus direct comparison between IHC and estimated cell type is not feasible, on average, the estimates are expected to reflect the IHC-based proportion of each cell type. MGP-based estimates were excluded from this analysis since the method outputs the relative abundance of each cell type across the subjects, rather than across the cell types. The median estimated proportion of neurons, astrocytes, and oligodendrocytes produced by the three applicable methods was of similar magnitude and comparable to the proportions expected based on IHC data (Additional file 1: Fig. S1b). In contrast, the median estimated proportion of endothelial and microglia cells was vastly dissimilar between the three estimation methods and different from the proportions expected based on IHC data. Most strikingly, the estimated proportion of both cell types based on CIBERSORT was 0, despite the IHC proportions of 0.1–0.2. This discrepancy cannot be explained by the fact that different individuals were included in our study and Patrick et al [15], since similar discordance was observed by Patrick et al. using IHC and RNA-seq data from the same individuals [15]. These results indicate that despite its popularity (> 4500 citations) and superior performance based on simulated/pseudo bulk brain tissue data [11], CIBERSORT is inadequate for analysis of real brain tissue data. Since dtangle and Bisque generally produced highly concordant estimates, for the rest of the paper we chose to focus on Bisque and MGP methods as representatives of two alternative approaches for transcriptomics-based estimation of cellular composition.

### Neighbouring brain tissue samples exhibit substantial interindividual variation in cell composition

We next sought to assess the interindividual variability in the estimated cell composition of neighbouring tissue samples from the same Brodmann area. For this purpose, we leveraged the data available from 42 individuals for which RNA-seq was carried out twice in two different (but neighbouring) tissue samples from the same Brodmann area, and seven individuals for which the same tissue sample RNA extract was sequenced twice (Fig. 1c, Additional file 1: Table S2). This setting allowed us to compare the transcriptomics-based estimates derived from the same or different tissue samples while accounting for the variation induced by technical factors arising during library preparation and sequencing. For reasons described above, cellular abundance was estimated using MGP and Bisque methods only.

**Fig. 2:**
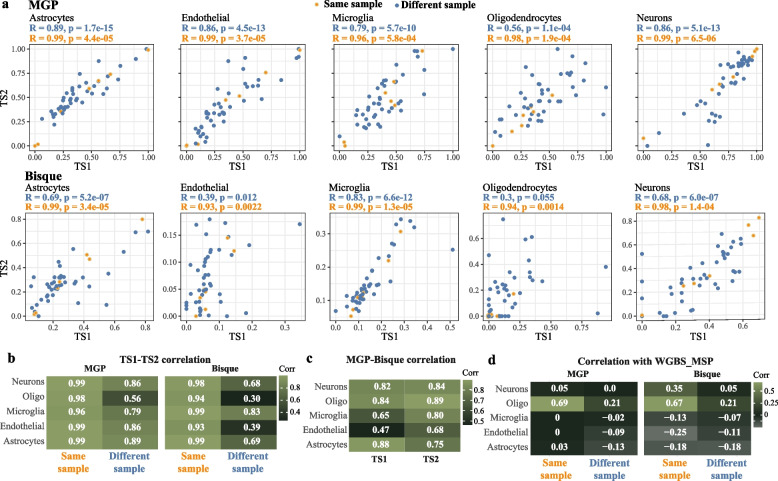
Estimate correlation. **a** Correlation between RNAseq1 (TS1) and RNAseq2 (TS2)-based estimates, using MGP (top) or Bisque (bottom). Each point represents one individual. For 42 of the individuals, mRNA was extracted for different but nearby tissue samples (blue), while for seven of the individuals the RNA was extracted from the same tissue sample but sequenced twice as part of RNAseq1 and RNAseq2 datasets (yellow). Pearson’s correlation for each cell type is shown separately for estimates derived from the same and different tissue samples. **b** Summary table of the correlation values shown in **a**. **c** Correlation between the estimates derives using the two RNA-based estimation methods in RNAseq1 (TS1) and RNAsq2 (TS2) datasets. **d** Correlation between RNA-based (MGP or Bisque) and WGBS-based estimates derived from the same (RNAseq1, WGBS) or different tissue samples (RNAseq2, WGBS). RNAseq2 estimates derived from the seven resequenced samples were excluded from the analysis

Regardless of the estimation approach (MGP or Bisque), estimates derived from the same tissue samples were highly correlated between the two RNA-seq datasets for all cell types (Pearson’s correlation 0.94–99, Fig. 2a,b). In contrast, the correlations between estimates derived from data of nearby tissue samples varied both between methods and cell types and were substantially lower than the correlations observed for estimates derived from the same tissue sample (Fig. 2a,b). The lowest correlation was observed for oligodendrocytes (Pearson’s correlation, MGP: 0.56, Bisque: 0.30), reflecting the expected variation in grey/white matter proportion among samples.

### Correlation is low between methods based on different omics modalities

We next assessed the correlation between estimates derived using different estimation methods and different omics modalities. The correlations were assessed between estimates based on RNA-seq (MGP and Bisque) and WGBS (WGBS_MSP and CETS [3], online methods), since for these omics modalities we had access to data extracted from the same tissue sample (Fig. 1c., Methods).

**Fig. 3:**
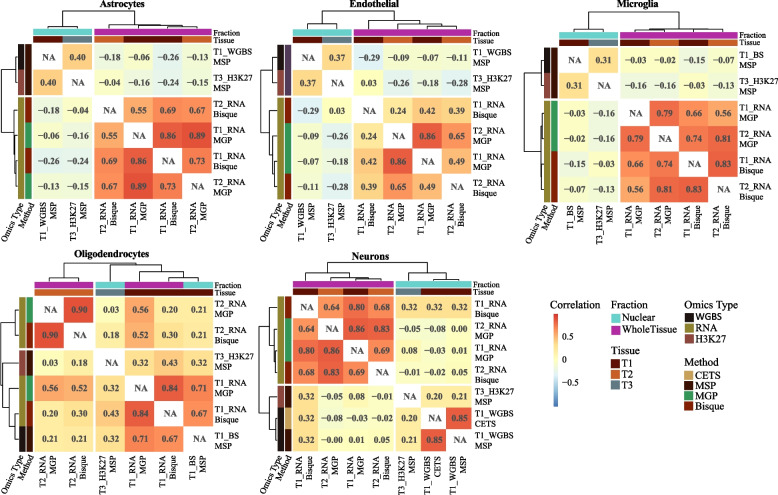
Estimate correlation across all estimation methods and data types. Clustering of the cell type estimates based on Pearson’s correlation between different estimation methods, omics type and tissue collections. Estimates from the seven resequenced individuals were excluded from this analysis. Omics type - omics data from which the estimates were derived; Fraction - tissue fraction captured by the omics data; Tissue - tissue collection from which the data was derived

We observed a high correlation between estimates based on the same omics data modality (MGP *vs.* Bisque-based estimates, IQR (*r*): 0.70–0.84, Fig. 2c; CETS *vs.* neuronal WGBS_MSP, *r* = 0.87). In contrast, when we compared transcriptomics-based and WGBS-based estimates derived from the same tissue extracts (Fig. 1c, Additional file 1: Table S2), we observed minimal to no correlation between the methods (IQR − 0.11–0.09), with the single exception of oligodendrocytes, for which moderate correlation was observed (*r*_*MGP*_ = 0.69, *r*_*Bisque*_ = 0.67, Figs. 2d and 3).

### Estimation methods cluster based on the tissue fraction they capture

As a final step, we looked at the correlations between all estimation methods. To this end, we excluded the seven samples which had been sequenced twice, since these would artificially increase the correlation between estimates derived from datasets RNAseq1 and RNAseq2. Clustering of the estimates indicated that estimates derived from nuclear omics modalities (WGBS and H3K27ac) are more similar to each other than to transcriptomics-based estimates derived from whole tissue omics data. This was true regardless of the method and of whether the estimates were derived from the same or different tissue sample (Fig. 3).

### Impact of the choice of estimation method of differential analysis of cell composition confounded samples

**Fig. 4:**
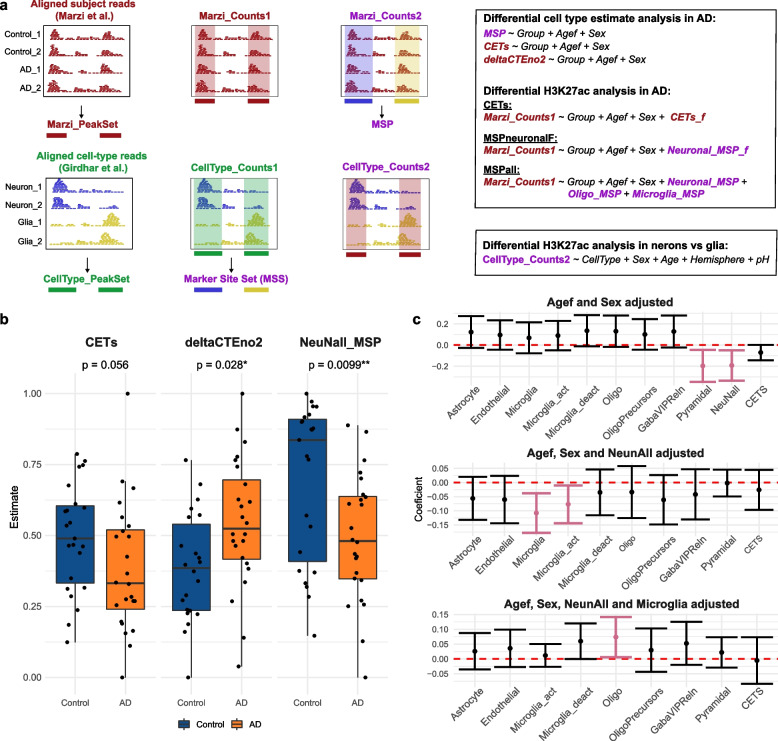
Experimental design of the re-analysis of Marzi et al. data. **a** Schematic representation of the two different ChIP-seq datasets (Marzi et al. and Girdhar et al.) used for the re-analysis and their respective peak sets (left), the original count matrices (middle) and the count matrices obtained by quantifying the reads from one data in the peak set from the other data (right). Data obtained from Marzi et al. are indicated in dark red. The different types of analyses and the different models used throughout the re-analysis are shown in the two frames. **b** Group differences in the neuronal estimates. The indicated *p*-values are based on the statistical models of differential cell type analyses described in **a**. **c** Estimated group effects (AD vs. control) and 95% confidence intervals of the indicated cell types. Shown are the estimates based on model adjusting for demographic covariates only (top), demographic covariates + neuronal MSP (middle) and demographic covariates + neuronal MSPs + microglia MSPs (bottom). Microglia_act: genes upregulated in activated microglia; Microglia_deact: genes downregulated in activated microglia; OligoPrecursors: oligodendrocyte precursor cells; GabaViPReln: VIP and Reelin-positive cells; NeuNall: All peaks with log2 fold change > 3 and adjusted *p*-value < 0.05 between neuronal (NeuN^+^) and glial (NeuN^−^) cells

To get an insight on the impact of the choice of estimation approaches on data analysis, we utilized publicly available H3K27ac ChIP-seq data from entorhinal cortex of healthy subjects and individuals with AD [14]. Since entorhinal cortex exhibits severe neuronal loss already at the early stages of the disease [34, 35], the authors adjusted their analysis for estimated neuron/glia ratio of the samples using CETS, calculated based on methylation data from neighbouring tissue sample from the same individuals. In spite of this adjustment, the differentially acetylated regions clustered the samples according to their CETS estimates rather than the disease state [14], indicating that the statistical model did not sufficiently account for the neuronal loss in AD. Based on our findings of high interindividual variability in cell composition of brain tissue samples (Fig. 2), we hypothesized that this may be at least partially caused by ChIP-seq and cell estimation analyses being carried out in different tissue homogenates (rather than aliquots of the same tissue sample homogenate). We thus re-analysed the data adjusting for cell type estimates calculated directly from the ChIP-seq data using MSPs. In order to restrict the difference between the original work and the re-analysis to estimates only, we used the count matrix and the BED file provided by the authors (GSE102538). The workflow of the re-analysis is illustrated in Fig. 4a.

### Cell type estimations based on MSPs, but not CETS, recapitulate the expected differences in cellular composition between individuals with AD and controls

We first examined whether CETS or MSPs reliably recapitulate the expected severe neuronal loss in the entorhinal cortex of individuals with terminal AD. In addition, we evaluated whether the decrease in neuronal abundance can be adequately captured by the transcript level of the neuronal marker *ENO2*, which was measured by the authors using real-time PCR (deltaCTEno2). The AD/control group difference for each type of estimates (CETS, neuronal MSPs, and deltaCTEno2) was assessed by linear models, adjusting for sex and age (represented as ordered factor, in compliance with the original analysis [14]). While all three measures exhibited the expected direction of change (decrease in CETS and neuronal MSPs, and increase in deltaCTEno2 in the AD group), this difference did not reach significance for CETS (*p* = 0.056, Fig. 4b). The difference observed for neuronal MSPs was highly significant and more pronounced than the difference observed for deltaCTEno2 (*p* = 9.9 × 10^−3^ *vs. p* = 0.028, Fig. 4b).

**Fig. 5:**
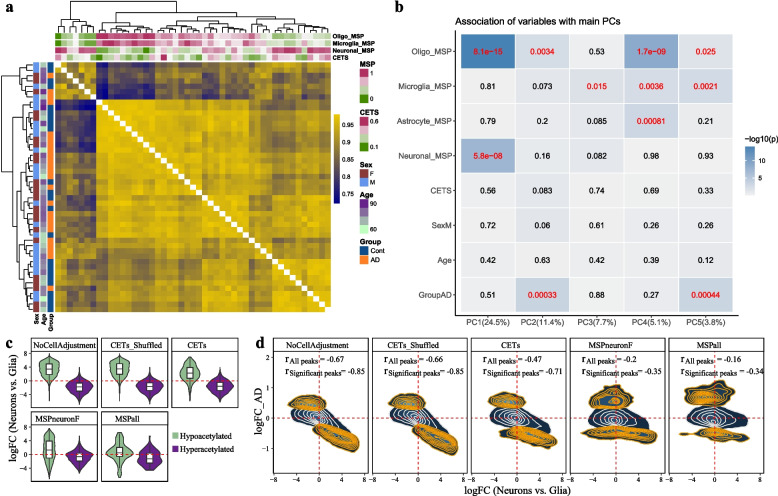
Impact of different cellular composition estimates on the analysis of H3K27ac ChIP-seq data. **a** Hierarchical clustering of the samples based on sample-sample correlation indicates that samples cluster according to their cellular composition, estimated by MSPs. **b** Association of demographic variables or cell estimates with the first five principal components of the data. The indicated numbers show the *p*-values of variables’ beta coefficients (*y*-axis) with the first five PCs (*x*-axis). **c,d** Comparison of the Marzi et al. data analysis output based on the different models, with cell type-specific H3K27ac ChIP-seq data from Girdhar et al. [25]. **c** H3K27ac fold of change in neurons vs glia in regions hypo- or hyperacetylated in AD based on each model. **d** Density plots of the correlation between the estimated H3K27ac effect sizes based on each model with the H3K27ac effect size in neurons compared to glial cells. Contours of the differentially acetylated regions based on each model are indicated in orange

In addition to the extensive neuronal loss, changes in additional cell types, e.g. reduction in microglia, has been shown to take place in the hippocampus and entorhinal cortex of individuals with AD [36, 37]. Thus, we investigated whether the MSP approach can detect alterations in additional cell types. Since estimates of different cell types are not independent of each other but rather exhibit co-linearity, we performed a step-wise regression analysis to identify cell types independently changing with the disease. This analysis indicated a decrease in neuronal and microglia MSPs and an increase in oligodendrocyte MSPs in individuals with AD (Fig. 4c). In addition to being corroborated by IHC-based studies [36, 37], both the decrease in microglia MSPs and the increase in oligodendrocyte MSPs are supported by a recent single-cell study of entorhinal cortex from individuals with AD [38].

### H3K27ac ChIP-seq data cluster samples based on cellular composition as estimated by MSPs

We next assessed the contribution of CETS, MSPs, and demographic variables provided by Marzi et al. [14] to the variance in the ChIP-seq data. Hierarchical clustering based on pairwise sample correlation of the ChIP-seq data indicated that the samples cluster mainly based on oligodendrocyte and neuronal MSPs (Fig. 5a). Accordingly, the first principal component of the data was significantly associated with these two MSPs, while the 3rd–5th principal components were associated with microglia MSP (Fig. 5b). None of the first five principal components (explaining 52% of the variance) was associated with CETS.

### CETS adjustment does not sufficiently account for neuronal loss in AD

Subsequently, we tested to what extent model adjustment using different approaches for estimation of cell type abundance is sufficient to account for neuronal loss in AD. We postulated that insufficient adjustment would result in neuron-enriched H3K27-acetylated regions to be identified as hypoacetylated in AD samples, while glia-enriched H3K27-acetylated regions will appear hyperacetylated. For these analyses, we quantified H3K27ac ChIP-seq reads from human NeuN^+^ (neurons) and NeuN^−^ (glia) cells [25], mapped to regions defined as peaks in Marzi et al., and carried out differential acetylation analysis between the two cell types (Fig. 4a). Next, we compared the cell type enriched H3K27ac regions with the differentially acetylated regions based on: (1) CETS-adjusted model (CETS model, similar to Marzi et al.) (2) model adjusted for neuronal MSPs and converted to ordered factor with five levels, similarly to the CETS model (MSPneuron) and (3) model adjusted for neuronal, microglia and oligodendrocyte MSPs, treating MSPs as continuous variables (MSPall model). While we believe that the MSPall model is more appropriate given that all three cell types exhibited group differences, the MSPneuron model allowed for direct comparison between CETS and neuronal MSPs. The outputs from the three models were compared to the output from a model without cell type adjustment (NoCellAdjustment), or a model in which the CETS estimates were randomly shuffled across the subjects within each group (CETS_Shuffled).

In concordance with the expected cell composition bias between AD and control samples, the analysis output based on NoCellAdjustment and CETS_Shuffled models mainly represented cell type-specific H3K27ac regions: 95% of the regions identified as hypoacetylated in AD corresponded to regions hyperacetylated in neurons, whereas 87% of the regions identified as hyperacetylated in AD corresponded to regions hypoacetylated in neurons (Fig. 5c). The strong bias towards cell type-specific H3K27ac regions was only slightly attenuated with the CETS model: 85% of the regions found significantly hypoacetylated in AD are hyperacetylated in neurons, while 83% of the regions found significantly hyperacetylated in AD are hypoacetylated in neurons (Fig. 5c). The cell type bias was substantially attenuated in the MSPneuron and MSPall model (hyperacetylated regions: 65.6 and 66.0%, respectively; hypoacetylated regions: 64.1 and 63.6%, respectively, Fig. 5c). Furthermore, a strong negative correlation between the H3K27ac fold of change in AD *vs*. controls and the fold of change in neurons *vs.* glia was observed for NoCellAdjustment and CETS_Shuffled models (Pearson’s correlation all regions: *r* =  − 0.67, significant regions: *r* =  − 0.85, both models, Fig. 5d). This correlation was moderately attenuated in the CETS model and substantially attenuated in both MSPneuron and MSPall models (Fig. 5d).

**Fig. 6:**
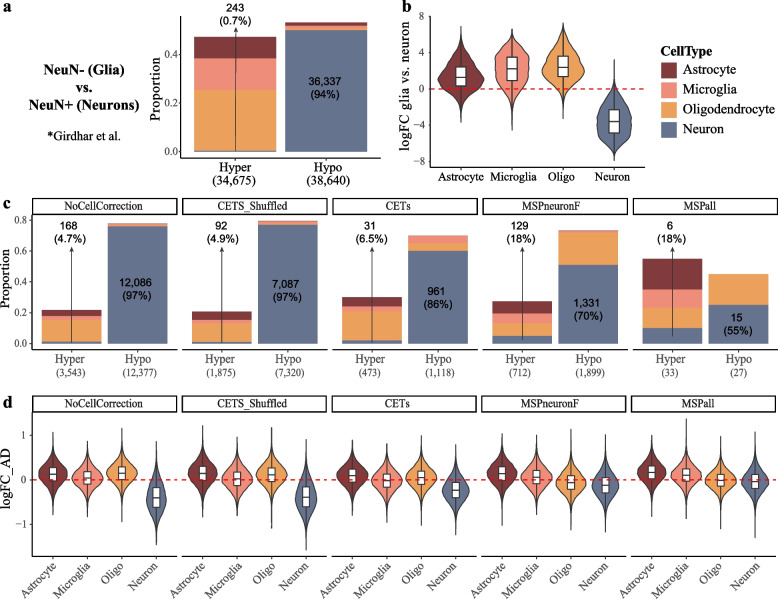
Assessment of the estimate performance using cell type-specific promoter/enhancer regions. Association between cell type-specific promoter/enhancer regions defined in Nott et al. [30], with the regions exhibiting differential acetylation between glia and neurons (**a**,**b**) or the outcome of differential acetylation analyses in AD using different cell composition estimation approaches (**c**,**d**). **a**,**b** Analysis of pooled glia (NeuN^−^) and neuron (NeuN^+^) H3K27ac ChIP-seq data from Girdhar et al. [25]. **c**,**d** Analysis of H3K27ac ChIP-seq data from bulk tissue entorhinal cortex of individuals with AD and controls from Marzi et. al [14]. **a**,**c** Stacked bar plots showing the proportion of differentially acetylated regions (FDR < 0.05) overlapping with promoter/enhancer regions unique to each of the cell types. Proportion (*y*-axis) was calculated relative to all significant regions overlapping with a cell type-specific promoter/enhancer region. The total number of hypo- and hyperacetylated regions meeting this criterion is indicated on the *x*-axis, in parenthesis. The number of peaks overlapping with neuron-specific promoter/enhancer regions is indicated on the bar plot area. Percentage was calculated separately for hypo- and hyperacetylated regions. **b**,**d** Violin plots showing the estimated acetylation fold of change in all peaks overlapping with a cell type-specific promoter/enhancer region, regardless of their significance level

To confirm our findings, we repeated the cell type enrichment analysis using the recently described enhancer and promoter regions of neurons, astrocyte, microglia and oligodendrocyte cells [30]. We first assessed whether differentially acetylated regions in glia compared to neuron cells [25] indeed overlap with cell type-specific promoter/enhancer regions. As expected, 94% of the differentially hypoacetylated regions in glia *vs*. neurons overlapped with neuron-specific promoter/enhancer regions, while overlap was only observed for 0.7% of the glia-hyperacetylated regions (Fig. 6a). Concordantly, when we assessed all the regions overlapping with cell type-specific promoter/enhancer region, the vast majority of neuronal promoter/enhancer regions (> 99.3%) were hypoacetylated in glia compared to neurons, while the opposite was true for glial promoter/enhancer regions (Fig. 6b).

In the AD dataset, 52, 50, 38, 38 and 29%, out of the differentially acetylated regions identified based on NoCellAdjustment, CETS_Shuffled, CETS, MSPneuron and MSPall models, respectively, overlapped with a unique cell type-specific promoter/enhancer region (Additional file 1: Table S3). Assessment of these regions confirmed that the CETS model does not sufficiently adjust for the major neuronal loss in entorhinal cortex in AD (Fig. 6c). Specifically, 86% of testable hypoacetylated regions in AD identified using CETS_model overlapped with a neuron-specific region (Fig. 6c). In sharp contrast, an overlap with unique neuronal enhancer/promoter regions was observed for only 6.5% of the testable hyperacetylated regions. This bias was similar to that observed for the NoCellAdjustment and CETS_Shuffled models (Fig. 6c). The cell type bias was substantially smaller in both the MSPneuron and MSPall models (Fig. 6c). Concordant associations were observed when all overlapping regions were included in the analysis and the fold of change in AD compared to controls in each of the cell type-specific enhancer/promoter regions was assessed (Fig. 6d).

## Discussion

In the absence of brain tissue datasets combining direct cell enumeration with omics-based estimates in the same sample, current methods for estimation of cell composition from bulk brain tissue data remain unvalidated. Thus, it remains unknown whether cell composition estimates used for adjustment of brain omics data are indeed reliable surrogates of the cellular composition of the samples. This can be highly detrimental, since the implementation of cell composition estimation methods with sub-optimal performance can falsely lead the researcher into believing that they have successfully accounted for the cell composition component in their analyses, and thus be drawn into the wrong conclusions.

In the current work, we assessed the correspondence between estimates of bulk brain tissue cell composition derived using different estimation methods, based on different omics modalities, and implementing different algorithms from the same or neighbouring tissue samples. Several key points arise from our work. First, cell composition can vary drastically even between two closely adjacent brain tissue samples from the same region. This can be explained by technical biases introduced during dissection of the sample, such as different proportions of white/grey matter and blood vessels, and/or uneven distribution of pathological changes, such as plaques or lesions. The implication of this finding is that estimates and even experimental cell counts from nearby tissue sample should not be used as surrogates for the cellular composition of bulk tissue samples subjected to omics analyses.

Second, as also demonstrated by our previous work [1, 2], different estimation methods based on the same transcriptomics data generally produce highly correlated estimates. The fact that this observation stems from fundamentally different methods, both in terms of the choice of cell type markers and the complexity of their underlying algorithms, implies that more sophisticated estimation methods do not offer substantial improvement over simpler approaches. Moreover, it strongly suggests that the currently employed “proof of concept” validation and assessment approaches are not sufficient to adequately evaluate the performance of estimation methods in the human brain.

Third, estimates based on whole brain tissue (transcriptomics) produce vastly different results than estimates based on only the nuclear fraction (DNA methylation, ChIP-seq). While this finding is based on a single dataset, the relatively large sample size and similar observations obtained for the different cell types suggest that it is very likely to be generalizable. We suggest that this discrepancy is caused by the fact that the vast portion of brain tissue is occupied by neuropil (i.e. dendrites, axons, synapses, glial cell processes and microvasculature) rather than cell nuclei (Fig. 1a). The signal from neuropil is captured in bulk tissue transcriptomics data and, therefore, impacts the transcriptomics-based estimates, introducing noise. Moreover, since neuropil comprises neuronal projections from distant neurons, often originating from an entirely different brain region and neuronal type [39, 40], estimates based on transcriptomics can be compromised by the current methodological assumptions. Namely, currently, cell type marker genes used in transcriptomics-based estimation approaches are selected based on their specificity in relation to other cell types residing in the same region. A major caveat in this approach is that a marker deemed to be specific based on this criterion might also be expressed in the synaptic terminals of neuronal populations that are not resident, but rather project to the same area. Inclusion of such markers will introduce noise into the estimation process, potentially biasing the outcomes. For example, striatal neurons are densely innervated by dopaminergic terminals, which contain transcripts overlapping with some of the neuron-specific markers in the striatum. Thus, unless the overlapping transcripts are excluded, variation in the state of striatal innervation, (e.g. due to parkinsonism) will impact the transcriptomics-based estimation of native striatal neuronal populations.

Lastly, our work demonstrates that, while including appropriate estimates of confounded cell types in statistical models can improve the outcome and interpretability of the analyses, it does not fully eliminate the confounding signal. Thus, great caution should be exercised when interpreting bulk tissue data from samples where major differences in cell composition are expected between the groups being compared. In fact, our findings suggest that such comparisons can lead to misleading conclusions and should be avoided.

One limitation of our work is that we only focused on a small fraction of the existing estimation approaches. The tested approaches, however, were chosen to include both older and simpler (MGP [9] and CIBERSORT [7]) and more recent and cutting edge approaches (Bisque [10] and dtangle [33]), which were demonstrated by to outperform other methods in the field [11]. Thus, our observations and very likely to generalize and our conclusions remain unchanged.

Our work raises the pertinent question—what is a brain cell, and particularly a neuron, in the context of bulk tissue analyses? Is it the nuclei, the cell bodies, the projections, or all of the above? Without addressing this conundrum, the task of “adjusting for cell composition” cannot be accomplished. Indeed, even if direct cellular enumeration from the same brain tissue sample becomes feasible, the architectural complexity of neurons will still present a challenge. The “gold standard” methods like IHC or Fluorescence-activated Cell Sorting (FACS) can only quantify cell bodies, or as in the case of human brain tissue, merely nuclei. Similarly, high-throughput single-cell approaches can only provide information regarding the number of cell bodies/nuclei. This type of enumeration would be missing a major component of the data and would not be able to adjust for differences in the tissue composition due to changes in synaptic density. This limitation is particularly relevant when working with aging [41, 42] or neurodegenerative disorders [43, 44], where the density of inbound synapses is substantially altered, even in areas with relatively preserved neuronal populations [45–48]. Importantly, alterations in synaptic density would have implications for non-neuronal estimates too. This is because the identical amount of starting material used for data generation implies that decrease in one tissue component (e.g. synaptic mRNA or protein) will necessarily result in over-representation of other components (e.g. glial mRNA or proteins).

## Conclusions

To summarize, this work highlights the caveats associated with cell type estimation approaches in brain tissue data and demonstrates the inadequacy of the currently employed validation approaches. While there is no doubt that adjusting for cell composition is crucial for analysis of bulk tissue data, great caution should be taken when the analyses involve brain tissue. Moreover, it should not be assumed that adjustment for cell composition estimates (or even experimental cell counts, if and when these become available) can fully account for cell composition when it is severely confounded with the condition of interest.

## Supplementary Information

**Additional file 1:** **Table S1.** Estimation approaches. **Table S2.** Subject description. **Table S3.** DARs overlaps with cell type-specific promoter/enhancer regions. **Figure S1.** Comparison between all transcriptomics-based estimation methods. All 49 individuals were included in the analysis. a. Pearson’s correlation of cell type estimates based on each of the indicated methods with the remaining three methods. The correlations were assessed separately in each of the RNAseq datasets, and then combined into one plot. Each point represents a single correlation value. b. Estimated proportion of each cell type based on ether of the two RNAseq datasets, in comparison to the expected proportion based on IHC data dorsolateral prefrontal cortex of individuals with Alzheimer’s disease and controls^9^.

**Additional file 2.**

## Data Availability

The data and the code to generate all the analyses and figures presented in this work can be accessed through the github repository: https://github.com/ltoker/Cellephant [19].

## Abbreviations

RNA-seq: RNA sequencing
ChIP-seq: ChIP sequencing
WGBS: Whole genome bisulfite sequencing
PFC: Prefrontal cortex
MGP: Marker Gene Profile
MSP: Marker Site Profile
IHC: Immunohistochemistry
CPM: Counts per million
RiP: Reads in peaks
DAR: Differentially acetylated regions
IQR: Interquartile range
AD: Alzheimer’s disease

## References

[1] Toker L, Mancarci BO, Tripathy S, Pavlidis P (2018). Transcriptomic evidence for alterations in astrocytes and parvalbumin interneurons in subjects with bipolar disorder and schizophrenia. Biol Psychiatry.

[2] Nido GS (2020). Common gene expression signatures in Parkinson’s disease are driven by changes in cell composition. Acta Neuropathol Commun.

[3] Guintivano J, Aryee MJ, Kaminsky ZA (2013). A cell epigenotype specific model for the correction of brain cellular heterogeneity bias and its application to age, brain region and major depression. Epigenetics.

[4] Jaffe AE, Irizarry RA (2014). Accounting for cellular heterogeneity is critical in epigenome-wide association studies. Genome Biol.

[5] Toker L (2021). Genome-wide histone acetylation analysis reveals altered transcriptional regulation in the Parkinson’s disease brain. Mol Neurodegener.

[6] Yu Q, He Z (2017). Comprehensive investigation of temporal and autism-associated cell type composition-dependent and independent gene expression changes in human brains. Sci Rep.

[7] Newman AM (2015). Robust enumeration of cell subsets from tissue expression profiles. Nat Methods.

[8] Chikina M, Zaslavsky E, Sealfon SC (2015). CellCODE. A robust latent variable approach to differential expression analysis for heterogeneous cell populations. Bioinformatics.

[9] Mancarci BO (2017). Cross-laboratory analysis of brain cell type transcriptomes with applications to interpretation of bulk tissue data. eNeuro.

[10] Jew B (2020). Accurate estimation of cell composition in bulk expression through robust integration of single-cell information. Nat Commun.

[11] Sutton GJ (2022). Comprehensive evaluation of deconvolution methods for human brain gene expression. Nat Commun.

[12] Houseman EA (2012). DNA methylation arrays as surrogate measures of cell mixture distribution. BMC Bioinformatics.

[13] van den Oord EJCG, Xie LY, Tran CJ, Zhao M, Aberg KA (2021). A targeted solution for estimating the cell-type composition of bulk samples. BMC Bioinformatics.

[14] Marzi SJ (2018). A histone acetylome-wide association study of Alzheimer’s disease identifies disease-associated H3K27ac differences in the entorhinal cortex. Nat Neurosci.

[15] Patrick E (2020). Deconvolving the contributions of cell-type heterogeneity on cortical gene expression. PLoS Comput Biol.

[16] Murphy K B, Nott A, Marzi S J. CHAS, a deconvolution tool, infers cell type-specific signatures in bulk brain histone acetylation studies of brain disorders. Preprint at 10.1101/2021.09.06.459142.

[17] Alves G (2009). Incidence of Parkinson’s disease in Norway: the Norwegian ParkWest study. J Neurol Neurosurg Psychiatry.

[18] Guitton R (2022). Ultra-deep whole genome bisulfite sequencing reveals a single methylation hotspot in human brain mitochondrial DNA. Epigenetics..

[19] Toker, L. Cellephant. Github. URL: https://github.com/ltoker/Cellephant.

[20] Patro R, Duggal G, Love MI, Irizarry RA, Kingsford C (2017). Salmon provides fast and bias-aware quantification of transcript expression. Nat Methods.

[21] Frankish A (2019). GENCODE reference annotation for the human and mouse genomes. Nucleic Acids Res.

[22] Darmanis S (2015). A survey of human brain transcriptome diversity at the single cell level. Proc Natl Acad Sci USA.

[23] Kelley KW, Nakao-Inoue H, Molofsky AV, Oldham MC (2018). Variation among intact tissue samples reveals the core transcriptional features of human CNS cell classes. Nat Neurosci.

[24] Velmeshev D (2019). Single-cell genomics identifies cell type-specific molecular changes in autism. Science.

[25] Girdhar K (2018). Cell-specific histone modification maps in the human frontal lobe link schizophrenia risk to the neuronal epigenome. Nat Neurosci.

[26] Love MI, Huber W, Anders S (2014). Moderated estimation of fold change and dispersion for RNA-seq data with DESeq2. Genome Biol.

[27] Bioconductor Package Maintainer (2021). liftOver: Changing genomic coordinate systems with rtracklayer::liftOver. R package version 1.18.0, https://www.bioconductor.org/help/workflows/liftOver/.

[28] Liao Y, Smyth GK, Shi W (2019). The R package Rsubread is easier, faster, cheaper and better for alignment and quantification of RNA sequencing reads. Nucleic Acids Res.

[29] Robinson MD, McCarthy DJ, Smyth GK (2010). edgeR: a Bioconductor package for differential expression analysis of digital gene expression data. Bioinformatics.

[30] Nott A (2019). Brain cell type–specific enhancer–promoter interactome maps and disease-risk association. Science.

[31] Schilder BM, Humphrey J, Raj T (2022). echolocatoR: an automated end-to-end statistical and functional genomic fine-mapping pipeline. Bioinformatics.

[32] Lawrence M (2013). Software for computing and annotating genomic ranges. PLoS Comput Biol.

[33] Hunt GJ, Freytag S, Bahlo M, Gagnon-Bartsch JA (2019). dtangle: accurate and robust cell type deconvolution. Bioinformatics.

[34] Van Hoesen GW, Hyman BT, Damasio AR (1991). Entorhinal cortex pathology in Alzheimer’s disease. Hippocampus.

[35] Arendt T, Brückner MK, Morawski M, Jäger C, Gertz H-J (2015). Early neurone loss in Alzheimer’s disease: cortical or subcortical?. Acta Neuropathol Commun.

[36] Navarro V (2018). Microglia in Alzheimer’s disease: activated, dysfunctional or degenerative. Front Aging Neurosci..

[37] Astillero-Lopez V (2022). Neurodegeneration and astrogliosis in the entorhinal cortex in Alzheimer’s disease: stereological layer-specific assessment and proteomic analysis. Alzheimers Dement..

[38] Grubman A (2019). A single-cell atlas of entorhinal cortex from individuals with Alzheimer’s disease reveals cell-type-specific gene expression regulation. Nat Neurosci.

[39] Calabresi P, Picconi B, Tozzi A, Ghiglieri V, Di Filippo M (2014). Direct and indirect pathways of basal ganglia: a critical reappraisal. Nat Neurosci.

[40] Canetta S (2020). Differential synaptic dynamics and circuit connectivity of hippocampal and thalamic inputs to the prefrontal cortex. Cerebral Cortex Communications.

[41] Pakkenberg B, Gundersen HJ (1997). Neocortical neuron number in humans: effect of sex and age. J Comp Neurol.

[42] Loerch PM (2008). Evolution of the aging brain transcriptome and synaptic regulation. PLoS ONE.

[43] Roeper J (2018). Closing gaps in brain disease—from overlapping genetic architecture to common motifs of synapse dysfunction. Curr Opin Neurobiol.

[44] Sakai J (2020). Core Concept: how synaptic pruning shapes neural wiring during development and possibly, in disease. PNAS.

[45] Morrison JH, Baxter MG (2012). The aging cortical synapse: hallmarks and implications for cognitive decline. Nat Rev Neurosci.

[46] Akram A (2008). Stereologic estimates of total spinophilin-immunoreactive spine number in area 9 and the CA1 field: relationship with the progression of Alzheimer’s disease. Neurobiol Aging.

[47] Gcwensa NZ, Russell DL, Cowell RM, Volpicelli-Daley LA (2021). Molecular mechanisms underlying synaptic and axon degeneration in Parkinson’s disease. Front Cellular Neurosci..

[48] Henstridge CM (2018). Synapse loss in the prefrontal cortex is associated with cognitive decline in amyotrophic lateral sclerosis. Acta Neuropathol.

