# Supplementary material for: Not every estimate counts – evaluation of cell composition estimation approaches in brain bulk tissue data
Source: Genome Med. 2023 Jun 7;15:41. doi: 10.1186/s13073-023-01195-2 (PMC10245417; doi:10.1186/s13073-023-01195-2)
Supplement: Supplementary file 1 — Additional file 1: Table S1. Estimation approaches. Table S2. Subject description. Table S3. DARs overlaps with cell type-specific promoter/enhancer regions. Figure S1. Comparison between all transcriptomics-based estimation methods. All 49 individuals were included in the analysis. a. Pearson’s correlation of cell type estimates based on each of the indicated methods with the remaining three methods. The correlations were assessed separately in each of the RNAseq datasets, and then combined into one plot. Each point represents a single correlation value. b. Estimated proportion of each cell type based on ether of the two RNAseq datasets, in comparison to the expected proportion based on IHC data dorsolateral prefrontal cortex of individuals with Alzheimer’s disease and controls9. [file 13073_2023_1195_MOESM1_ESM.docx]

**Additional file 1**

|  | **Material** | **Marker source** | **Measured value** | **Reference** |
| --- | --- | --- | --- | --- |
| CETS | WGBS | Pooled cell data, human | Neuron/glia ratio (per individual) | ^1^ |
| Bisque | RNA | Single cell data, human | Proportion of each cell type (per individual) | ^2^ |
| dtangle | RNA | Single cell data*, human | Proportion of each cell type (per individual) | ^3^ |
| CIBERSORT | RNA | Single cell data*, human | Proportion of each cell type (per individual) | ^4^ |
| MGP | RNA | Pooled cell data, mouse | Relative abundance of each cell type across individuals (per cell type) | ^5,6^ |
| MSP | H3K27ac WGBS** | Pooled cell data, mouse | Relative abundance across individuals (per cell type) | ^7^ |

Table S1. Estimation approaches

* Using “MB” option from https://voineagulab.shinyapps.io/BrainDeconvShiny/ ^8^. The set of markers is based on expression data from multiple sources of human single cell data.

**The method was developed for H3K27ac ChIP-seq data and adopted for WGBS data, see online methods section

Table S2. Subject description

| BioBankID | Cohort | Condition | Age | Sex | PMI | RIN | Re-sequenced |
| --- | --- | --- | --- | --- | --- | --- | --- |
| NM-027 | NBB | PD | 86 | M | 5.5 | 6.4 | No |
| NM-059 | NBB | Control | 85 | F | 7 | 6.6 | No |
| NM-028 | NBB | PD | 84 | M | 1.2 | 8.7 | No |
| NM-060 | NBB | Control | 84 | F | 6 | 6.4 | No |
| NM-029 | NBB | PD | 84 | F | 7.5 | 7.2 | No |
| NM-061 | NBB | Control | 76 | F | 7.2 | 7.4 | No |
| NM-030 | NBB | PD | 80 | F | 7 | 6.8 | No |
| NM-062 | NBB | Control | 89 | F | 5.5 | 5.4 | No |
| NM-031 | NBB | PD | 81 | M | 4.5 | 7.2 | No |
| NM-063 | NBB | Control | 102 | M | 5 | 6.7 | No |
| NM-032 | NBB | PD | 77 | F | 7.2 | 8.1 | No |
| NM-064 | NBB | Control | 91 | F | 4.2 | 3.2 | No |
| NM-033 | NBB | PD | 84 | M | 5 | 8.7 | No |
| NM-065 | NBB | Control | 96 | M | 4.2 | 5.9 | No |
| NM-034 | NBB | PD | 75 | M | 6 | 8.9 | No |
| NM-066 | NBB | Control | 95 | M | 7.3 | 7.1 | No |
| NM-035 | NBB | PD | 88 | F | 6.5 | 5.9 | No |
| NM-067 | NBB | Control | 78 | F | 7.2 | 9.1 | No |
| NM-036 | NBB | PD | 75 | M | 8.5 | 6.1 | No |
| NM-068 | NBB | Control | 93 | M | 7.5 | 8 | No |
| NM-001 | PW | PD | 86 | M | 30 | 3.1 | No |
| NM-069 | NBB | Control | 73 | M | 8 | 3.7 | No |
| NM-002 | PW | PD | 88 | M | 30 | 5.1 | No |
| NM-047 | PW | Control | 88 | F | 50 | 5.6 | No |
| NM-006 | PW | PD | 72 | M | 48 | 5.6 | No |
| NM-040 | PW | Control | 87 | M | 40 | 5.9 | Yes |
| NM-009 | PW | PD | 95 | M | 48 | 5.9 | No |
| NM-043 | PW | Control | 87 | M | 40 | 6 | Yes |
| NM-005 | PW | PD | 69 | M | 57 | 4.5 | No |
| NM-039 | PW | Control | 86 | F | 72 | 4.1 | Yes |
| NM-008 | PW | PD | 82 | M | 84 | 6.1 | No |
| NM-046 | PW | Control | 85 | F | 72 | 5.9 | No |
| NM-004 | PW | PD | 81 | F | 15 | 5.4 | No |
| NM-037 | PW | Control | 79 | M | 48 | 3.4 | Yes |
| NM-003 | PW | PD | 82 | M | 46 | 7.2 | No |
| NM-041 | PW | Control | 66 | F | 48 | 5.1 | Yes |
| NM-007 | PW | PD | 78 | F | 24 | 6.2 | No |
| NM-045 | PW | Control | 66 | M | 87 | 3.6 | No |
| NM-010 | PW | PD | 90 | F | 24 | 6.6 | No |
| NM-050 | PW | Control | 65 | M | 48 | 4.9 | No |
| NM-012 | PW | PD | 72 | F | 72 | 5.9 | No |
| NM-042 | PW | Control | 63 | M | 24 | 6.5 | Yes |
| NM-013 | PW | PD | 74 | M | 24 | 5.3 | No |
| NM-044 | PW | Control | 63 | F | 48 | 6.1 | Yes |
| NM-011 | PW | PD | 80 | F | 48 | 4.1 | No |
| NM-015 | PW | PD | 86 | F | 60 | 6.9 | No |
| NM-017 | PW | PD | 83 | F | 30 | 4.2 | No |
| NM-016 | PW | PD | 85 | M | 30 | 5.8 | No |
| NM-014 | PW | PD | 69 | F | 30 | 3 | No |

PD - Parkinson’s disease; PMI - postmortem interval; Re-sequenced – indicates whether or not the same tissue sample was used for both RNAseq experiments

Table S3. DARs overlaps with cell type-specific promoter/enhancer regions

| **Direction**  **AD** | **Method** | **Total DAR** | **Cell**  **Type** | **N** | **Proportion_A** | **Proportion_B** |
| --- | --- | --- | --- | --- | --- | --- |
| **Hyperacetylated** | NoCellCorrection | 3543 | Astrocyte | 658 | 0.041 | 0.186 |
|  |  |  | Microglia | 418 | 0.026 | 0.118 |
|  |  |  | Oligo | 2299 | 0.14 | 0.649 |
|  |  |  | Neuronal | 168 | 0.011 | 0.047 |
|  | CETS_Shuffled | 1875 | Astrocyte | 500 | 0.054 | 0.267 |
|  |  |  | Microglia | 211 | 0.023 | 0.113 |
|  |  |  | Oligo | 1072 | 0.12 | 0.572 |
|  |  |  | Neuronal | 92 | 0.01 | 0.049 |
|  | CETs | 473 | Astrocyte | 96 | 0.06 | 0.203 |
|  |  |  | Microglia | 50 | 0.031 | 0.106 |
|  |  |  | Oligo | 296 | 0.19 | 0.626 |
|  |  |  | Neuronal | 31 | 0.019 | 0.066 |
|  | MSPneuronF | 712 | Astrocyte | 205 | 0.079 | 0.288 |
|  |  |  | Microglia | 166 | 0.064 | 0.233 |
|  |  |  | Oligo | 212 | 0.081 | 0.298 |
|  |  |  | Neuronal | 129 | 0.049 | 0.181 |
|  | MSPall | 33 | Astrocyte | 12 | 0.2 | 0.364 |
|  |  |  | Microglia | 7 | 0.12 | 0.212 |
|  |  |  | Oligo | 8 | 0.13 | 0.242 |
|  |  |  | Neuronal | 6 | 0.1 | 0.182 |
| **Hypoacetylated** | NoCellCorrection | 12377 | Astrocyte | 54 | 0.0034 | 0.004 |
|  |  |  | Microglia | 134 | 0.0084 | 0.011 |
|  |  |  | Oligo | 103 | 0.0065 | 0.008 |
|  |  |  | Neuronal | 12086 | 0.76 | 0.976 |
|  | CETS_Shuffled | 7320 | Astrocyte | 26 | 0.0028 | 0.004 |
|  |  |  | Microglia | 128 | 0.014 | 0.017 |
|  |  |  | Oligo | 79 | 0.0086 | 0.011 |
|  |  |  | Neuronal | 7087 | 0.77 | 0.968 |
|  | CETs | 1118 | Astrocyte | 1 | 0.00063 | 0.001 |
|  |  |  | Microglia | 78 | 0.049 | 0.070 |
|  |  |  | Oligo | 78 | 0.049 | 0.070 |
|  |  |  | Neuronal | 961 | 0.6 | 0.860 |
|  | MSPneuronF | 1899 | Astrocyte | 6 | 0.0023 | 0.003 |
|  |  |  | Microglia | 26 | 0.01 | 0.014 |
|  |  |  | Oligo | 536 | 0.21 | 0.282 |
|  |  |  | Neuronal | 1331 | 0.51 | 0.701 |
|  | MSPall | 27 | Oligo | 12 | 0.2 | 0.444 |
|  |  |  | Neuronal | 15 | 0.25 | 0.556 |

Total DAR – total number of DAR overlapping with cell-type specific promoter/enhancer. N - number of DARs overlapping with each cell type-specific promoter/enhancer region. Proportion_A - proportion of overlaps out of all overlapping DAR (both hypo- or hyper acetylated). Proportion_B - proportion of overlaps out of direction-specific overlapping DAR (either hypo- or hyper acetylated)


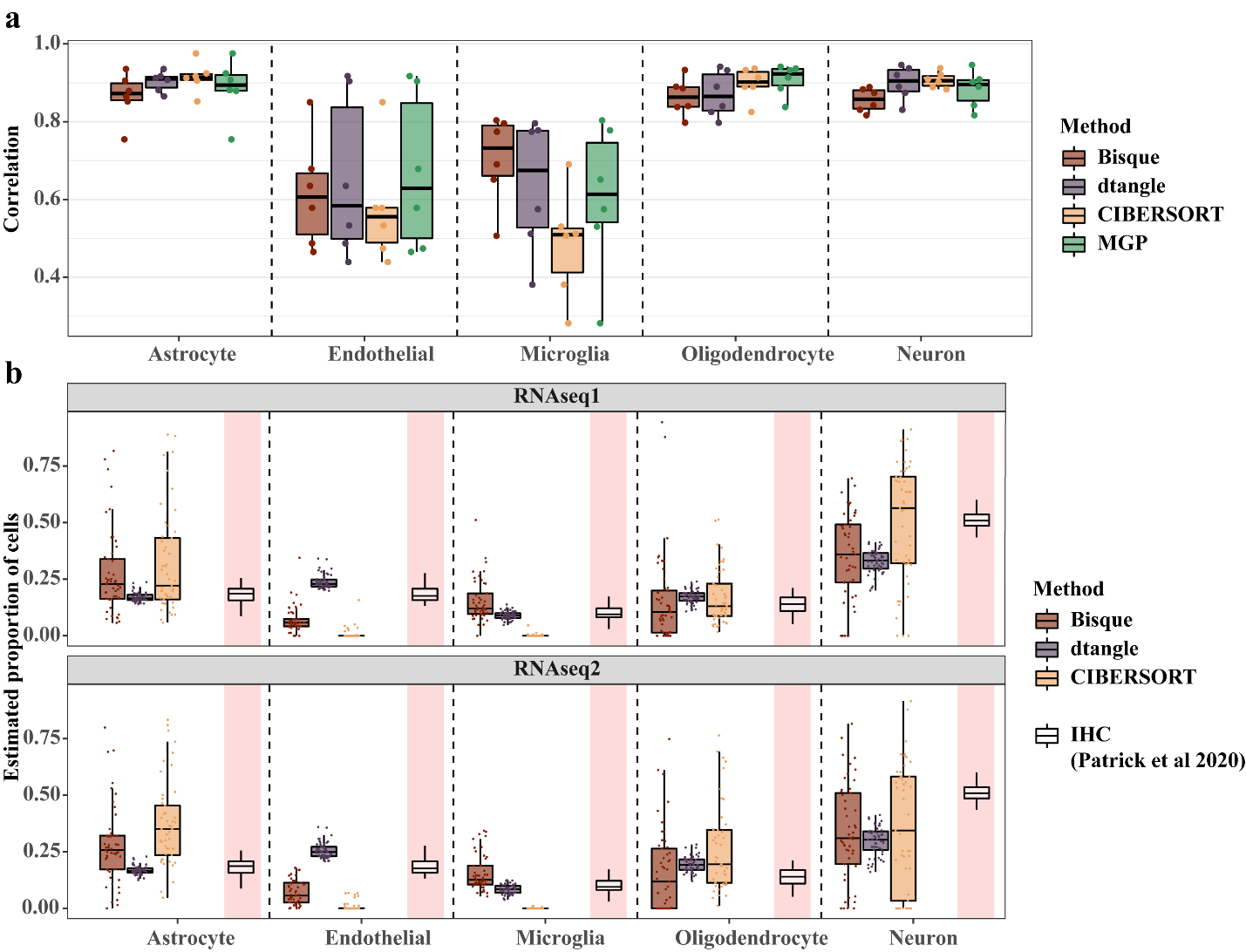


**Figure S1. Comparison between all transcriptomics-based estimation methods**

All 49 individuals were included in the analysis. **a.** Pearson’s correlation of cell type estimates based on each of the indicated methods with the remaining three methods. The correlations were assessed separately in each of the RNAseq datasets, and then combined into one plot. Each point represents a single correlation value. **b.** Estimated proportion of each cell type based on ether of the two RNAseq datasets, in comparison to the expected proportion based on IHC data dorsolateral prefrontal cortex of individuals with Alzheimer’s disease and controls^9^.

**References:**

1. Guintivano, J., Aryee, M. J. & Kaminsky, Z. A. A cell epigenotype specific model for the correction of brain cellular heterogeneity bias and its application to age, brain region and major depression. *Epigenetics* **8**, 290–302 (2013).

2. Jew, B. *et al.* Accurate estimation of cell composition in bulk expression through robust integration of single-cell information. *Nature Communications* **11**, 1971 (2020).

3. Hunt, G. J., Freytag, S., Bahlo, M. & Gagnon-Bartsch, J. A. dtangle: accurate and robust cell type deconvolution. *Bioinformatics* **35**, 2093–2099 (2019).

4. Newman, A. M. *et al.* Robust enumeration of cell subsets from tissue expression profiles. *Nat Methods* **12**, 453–457 (2015).

5. Mancarci, B. O. *et al.* Cross-Laboratory Analysis of Brain Cell Type Transcriptomes with Applications to Interpretation of Bulk Tissue Data. *eNeuro* ENEURO.0212-17.2017 (2017) doi:10.1523/ENEURO.0212-17.2017.

6. Toker, L., Mancarci, B. O., Tripathy, S. & Pavlidis, P. Transcriptomic Evidence for Alterations in Astrocytes and Parvalbumin Interneurons in Subjects With Bipolar Disorder and Schizophrenia. *Biol. Psychiatry* **84**, 787–796 (2018).

7. Toker, L. *et al.* Genome-wide histone acetylation analysis reveals altered transcriptional regulation in the Parkinson’s disease brain. *Molecular Neurodegeneration* **16**, 31 (2021).

8. Sutton, G. J. *et al.* Comprehensive evaluation of deconvolution methods for human brain gene expression. *Nat Commun* **13**, 1358 (2022).

9. Patrick, E. *et al.* Deconvolving the contributions of cell-type heterogeneity on cortical gene expression. *PLoS Comput Biol* **16**, e1008120 (2020).
